# Dataset for worldwide survey of cerebrospinal total protein upper reference values

**DOI:** 10.1016/j.dib.2019.103760

**Published:** 2019-03-07

**Authors:** Pierre R. Bourque, John Brooks, Jodi Warman-Chardon, Harald Hegen, Florian Deisenhammer, Chris R. McCudden, Ari Breiner

**Affiliations:** a Division of Neurology, Department of Medicine, The Ottawa Hospital and University of Ottawa, Ottawa, Ontario, Canada; b Ottawa Hospital Research Institute, Ottawa, Ontario, Canada; c Department of Neurology, Neuroimmunology Laboratory, Medical University of Innsbruck, Innsbruck, Austria; d Department of Pathology & Laboratory Medicine, The Ottawa Hospital and University of Ottawa, Ontario, Canada

## Abstract

This article reports data pertaining to a worldwide web-based survey referenced in the publication “Adult CSF Total Protein: Higher upper reference limits should be considered worldwide ” (P.R. Bourque, et al., 2019). This survey was distributed to corresponding authors of the journal Neurology and the Journal of neurological sciences for the period of Jan–Dec 2017. The response rate was 36.9%. Additional results were collated through networking and national associations. There were 473 unique responses from clinical hospital laboratories in 69 countries: North America 178, South America 26, Europe 139, Africa 20, Asia 102 and Oceania 8. The upper reference limit for cerebrospinal fluid total protein ranged from 0.2 g/L to 0.8 g/L. 86.8% of the survey responses were 0.45 g/L or less. Data is presented separately for tertiary/academic and non-university/community centers.

**Table:** Specifications table

Subject area	*Neurology and Laboratory Medicine*
More specific subject area	*Cerebrospinal fluid protein analysis*
Type of data	*Tables, bar graph, flow chart*
How data was acquired	*Web-based survey*
Data format	*Raw, analyzed, descriptive and statistical data*
Experimental factors	*Web-based survey on laboratory reference values for cerebrospinal fluid total protein, distributed to clinical neuroscientists*
Experimental features	*Cerebrospinal fluid reference limits were analyzed by geographical location and institution category*
Data source location	*Ottawa, Ontario, Canada*
Data accessibility	*Data is with article*
Related research article	Bourque PR, Breiner A, Moher D et al. Adult CSF total protein: Higher upper reference limits should be considered worldwide. A web-based survey. Journal of the neurological sciences. 2019; 396:48–51

**Table:** 

**Value of the data** • The data documents which CSF total protein reference values are currently used in 69 countries worldwide. These values can be contrasted with recently published rigorous laboratory studies and a systematic review [1], [2], [3], [4]. • The data illustrates and compares current practice patterns for CSF total protein reference limits in community and tertiary care institutions. • The data can be used by clinical biochemists and clinical neuroscientists in different geographic locations to reassess their institutional reference values.

## 1 Data

**Fig. 1:**
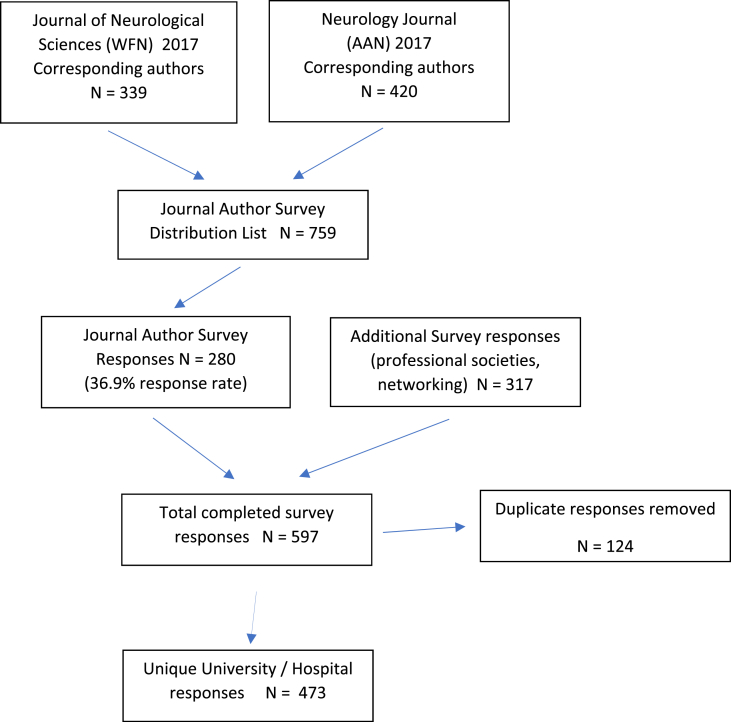
Flow chart of survey data acquisition. WFN: World Federation of Neurology AAN: American Academy of Neurology.

**Fig. 2:**
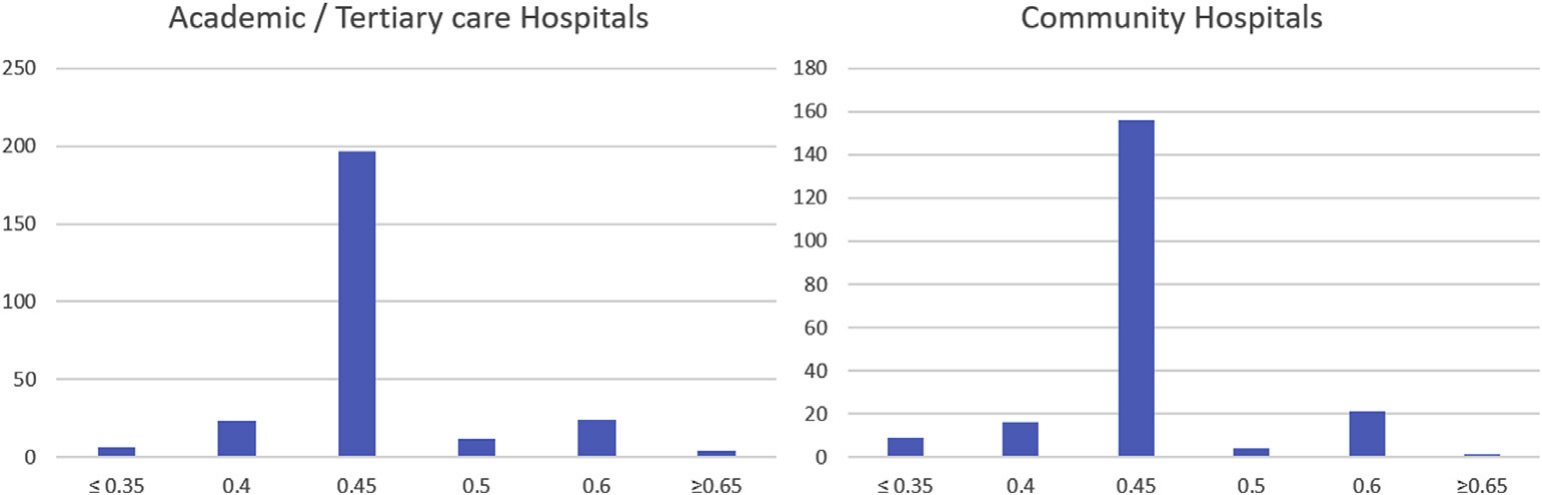
Histograms of CSF-TP upper reference values in Academic and Community institutions. The number of institutions is plotted on the y-axis, the CSF-TP (g/L) on the x-axis. The same median value of 0.45 g/L was obtained for both types of institutions.

**Fig. 3:**
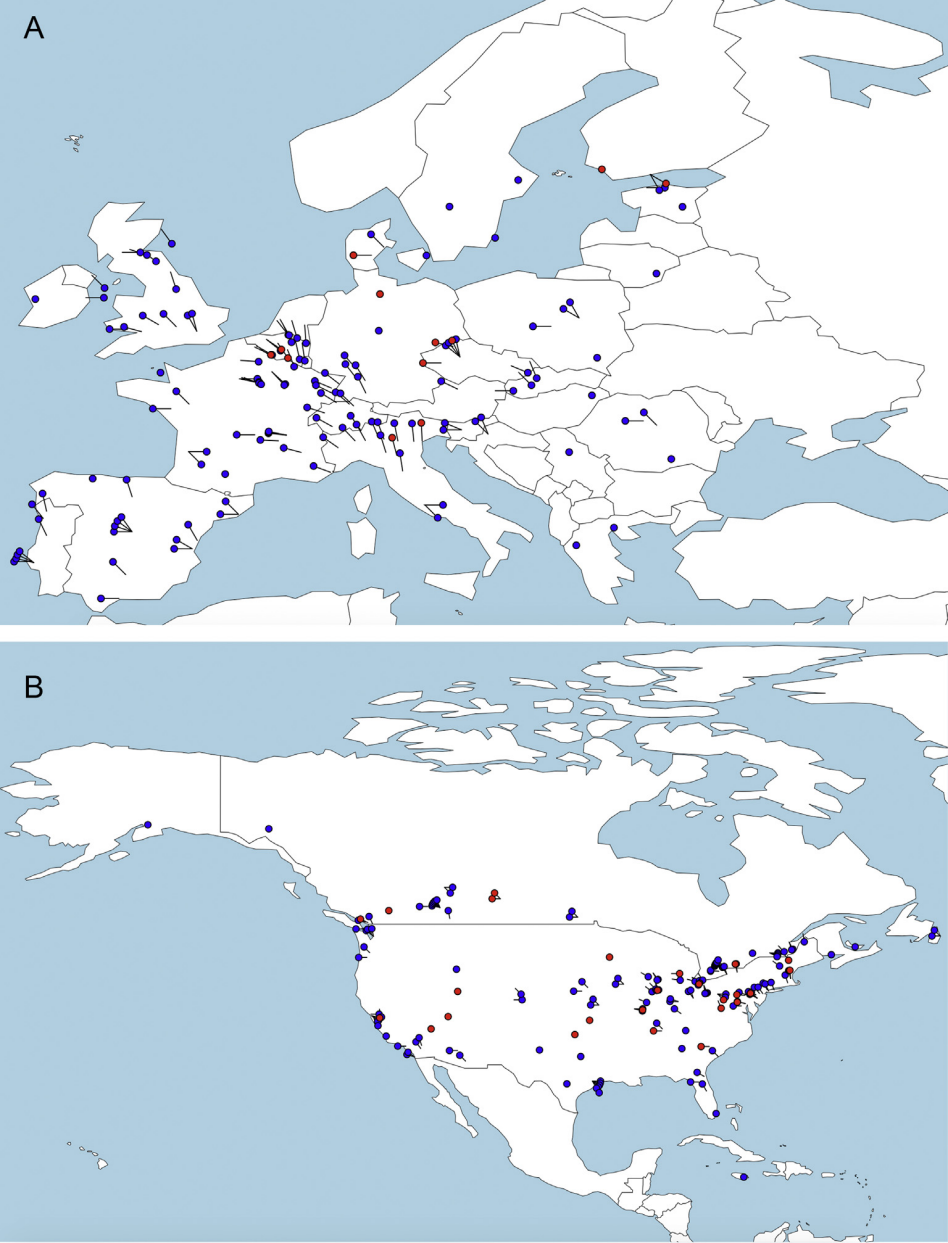

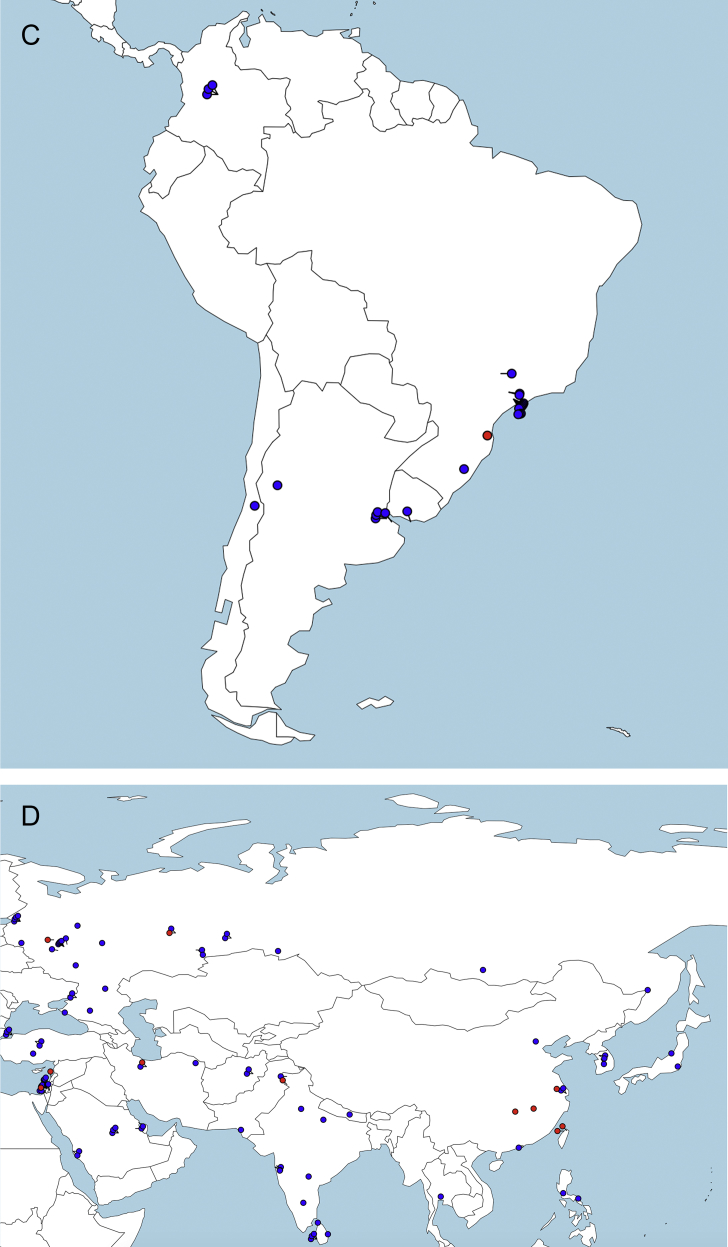

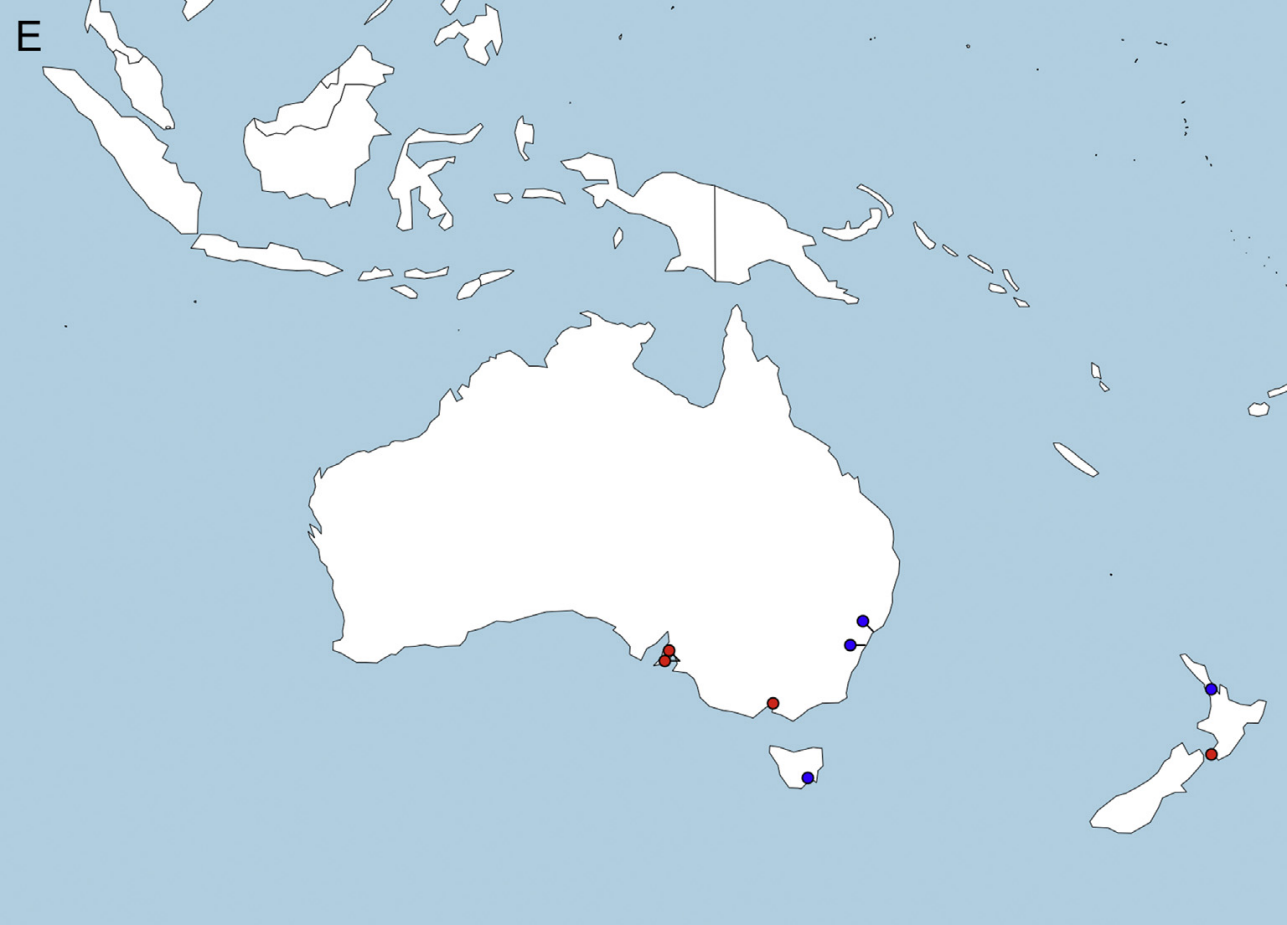
Continental geolocation maps of CSF TP responses from web-based survey. Hospital/Laboratory locations are represented in black for values of 0.45 g/L or less, and in red for values greater than 0.45g/L. A: Europe, B: North America, C: South America, D: Asia, E: Oceania.

A flow chart of the survey response acquisition process and exclusion of duplicate answers is presented in Fig. 1. Upper reference limit results for CSF total protein are presented in histograms, separated by type of clinical hospital laboratory (Fig. 2). A table provides data on the number of responses, institutional type, and CSF-TP range, divided by continent and country. Geolocation maps document the worldwide distribution of clinical hospital laboratories surveyed (Figs. 3A–E).

## 2 Experimental design, materials, and methods

This survey was approved by the Ethics board of the Ottawa Hospital Research Institute. To obtain a distribution list, we first generated a list of 759 clinical neuroscientists from the “corresponding author” designations in the following two clinical neurology journals, chosen for their worldwide catchment: Neurology (410 email addresses) and the Journal of Neurological Sciences (339 email addresses). Both journals were surveyed for the period of Jan–Dec 2017. Additional responses were obtained by distribution to a listserv approved by the relevant authority (Canadian Neurological Sciences Federation, Russian Federation of Laboratory Medicine). Finally, individual worldwide responses were added through direct networking with clinical neurology and laboratory medicine peers. When several responses were obtained from the same center, the highest stated value was entered for data analysis. As the data does not approximate a normal distribution, median CSF total protein values are reported for the academic centers and community institutions. Table 1.

**Table 1:** World distribution of institutional CSF-TP values, by country and academic/community affiliation.

	Total # Centers	# Academic (%)	CSF-TP > 0.45 g/L (All Centers)	CSF-TP > 0.45 g/L (Academic)	CSF-TP > 0.45 g/L (Community)
**World**	**473**	**270 (57.1%)**	**61/473 (12.9%)**	**47/270 (17.4%)**	**14/203 (6.9%)**
**N. America**	**178**	**113 (63.5%)**	**29/178 (16.3%)**	**16/113 (14.2%)**	**13/65 (20%)**
Canada	53	31 (58.5%)	5/53	3/31	2/22
USA	121	79 (65.3%)	24/121	13/79	11/42
**S. America**	**26**	**8 (30.8%)**	**2/26 (7.7%)**	**2/8 (25%)**	**0/18 (0%)**
Brazil	14	6 (42.9%)	1/14	1/6	0/8
**Europe**	**139**	**85 (61.2%)**	**25/139 (18.0%)**	**18/85 (21%)**	**7/54 (13%)**
France	25	15 (60.0%)	1/25	0/15	1/10
Italy	12	4 (33.3%)	1/12	0/4	1/8
Spain	12	10 (83.3%)	1/12	1/10	0/2
U. Kingdom	12	7 (58.3%)	2/12	1/7	1/5
**Asia**	**102**	**49 (48.0%)**	**11/102 (10.8%)**	**8/49 (16%)**	**3/53 (5.7%)**
India	10	6 (60.0%)	1/10	1/6	0/4
Israel	14	7 (50.0%)	1/14	1/7	0/7
Russia	30	7 (23.3%)	2/30	1/7	1/23
**Africa**	**20**	**13 (65.0%)**	**3/20 (15%)**	**0/13 (0%)**	**3/7 (42.9%)**
**Oceania**	**8**	**2 (25.0%)**	**4/8 (50%)**	**2/2 (100%)**	**2/6 (33.3%)**

Within continents, individual country results are presented only if there were 10 or more institutional replies. CSF-TP: Cerebrospinal Fluid Total Protein.

The survey consisted of the following 3 main questions: 1) What is the CSF TP upper reference value at your institution for adult patients? (e.g. < 0.45 g/L/ < 0.60 g/L/or specify any different value); 2) Do you use age-adjusted reference limits for adults (e.g. < 50 years, > 50 years of age) ? (yes/no); 3) What is the source of your CSF TP reference intervals? (Published laboratory references/Institution specific reference values, derived from local data/Unknown).
